# Community-Engaged Approach to Improve Food Access and Consumption of Fruits and Vegetables in a Rural Appalachian Community

**DOI:** 10.3390/nu17030431

**Published:** 2025-01-24

**Authors:** Courtney T. Luecking, Makenzie Barr-Porter, Dawn Brewer, Kathryn M. Cardarelli

**Affiliations:** 1 Department of Dietetics and Human Nutrition, University of Kentucky, Lexington, KY 40506, USA; makenzie.barr@uky.edu (M.B.-P.); dbr239@uky.edu (D.B.); 2 School of Public Health and Information Sciences, University of Louisville, Louisville, KY 40202, USA; kathryn.cardarelli@louisville.edu

**Keywords:** community–academic partnership, community advisory board, policy, system, and environment, Cooperative Extension, nutrition, intervention planning

## Abstract

**Background/Objectives**: Community–academic partnerships offer unique opportunities to leverage expertise and resources to modify structural factors that address community priorities. However, few in-depth examples of successful partnerships in rural communities to improve food security are available. This manuscript describes the process of building a partnership to reduce food insecurity in a rural Kentucky community. **Methods**: The research team worked with Cooperative Extension to form a community advisory board (CAB) representative of diverse sectors of the community who had interest in food security, agriculture, and/or nutrition. The CAB convened regularly, in-person or virtually, to review community assessment information and identify, select, and adapt relevant multi-level interventions. CAB members were invited to complete two surveys and participate in a listening session to provide feedback on working with academic partners. **Results**: Over the first two years of the project, 17 people served on the CAB. Early in the project, the CAB prioritized interventions for working families, relatives raising children, and lower income households. Some ideas were able to come to fruition (e.g., family cooking social classes, raised garden beds), while others were explored but were unable to gain momentum (e.g., mobile market) due to challenges with feasibility, organizational capacity, and/or interest. CAB members reported high engagement and strong communication between community and academic partners. **Conclusions**: Full exploration of potential solutions suggested by CABs may build trust between community and academic partners and could provide a strategic selection process for multi-level, evidence-based interventions deemed feasible for addressing complex issues such as food insecurity and nutritional health outcomes.

## 1. Introduction

Rural communities continue to experience significant health disparities in comparison to their urban counterparts [1,2]. Adverse differences in health are driven by several factors tied to rurality, including geographical isolation, lack of access to healthcare, and poor infrastructure for preventive medicine and interventions [3,4]. Disparities in obesity are particularly great between rural and urban populations [5,6]. While individual factors, such as health behavior, knowledge, self-efficacy, and skills (e.g., meal planning and cooking skills), play a role in healthy living, broader factors are at play for many rural residents [7,8]. Structural factors, such as policies and a community’s physical and social environment, directly impact resources available to residents [9]. For example, access to nutritious food, health promotion education, and community resources are often lacking in rural communities [10]. Environmental factors contributing to obesity inequities may be largely attributed to economic and built environment differences, and many of these challenges were exacerbated by the Covid-19 pandemic [7,11].

Ultimately, strategic initiatives that consider the heterogeneous nature of rurality are needed [2,12]. Multi-level programs that involve policy, systems, and environmental (i.e., upstream) approaches to address structural factors in combination with individually focused education may help prevent obesity and promote health equity [13]. However, adaptations are necessary for these approaches to be successful in rural environments [14,15,16]. Working with rural communities to identify and adapt possible obesity prevention solutions and to build capacity for sustained implementation will ensure strategies fit within the local context and can address equity issues [13,16,17]. Community–academic partnerships offer unique opportunities to share expertise and resources, build or modify systems, and disseminate and implement interventions that address community priorities [18,19].

In spring 2020, a team of researchers at the University of Kentucky began a multi-year effort to improve food security and nutritional health outcomes for low-income populations in a rural community. Researchers chose to work with and through the local Cooperative Extension Service (i.e., Extension). Extension is a nationwide network operated through Land-Grant Universities that provides a system for sustained dissemination and implementation of evidence-based and evidence-informed interventions tailored to local communities [20,21]. Extension is a well-respected and connected community entity, particularly in rural communities, that is often engaged in coalitions to improve community health [22,23].

Community advisory boards (CABs) are an effective way to build mutually beneficial partnerships between communities and universities [24,25,26]. Yet, few examples of the process of forming and engaging a CAB to foster community–academic partnerships in rural communities are available to researchers interested in community-engaged work [27,28]. This manuscript describes the process of building and refining a community–academic partnership through creation of a CAB to enhance healthy food access and reduce food insecurity in a rural Kentucky community. While findings focus on a single community, the detailed process and lessons learned will benefit other rural areas through providing a replicable model for building community partnerships to overcome common barriers for adapting and implementing policy, systems, and environmental changes in rural communities [14,29].

### Community Context

In Kentucky, approximately 40% of the population resides in rural locations [30]. Laurel County, the community of focus, is in the southeastern part of Appalachian Kentucky. The county’s population is primarily white, and 20.5% of families live in poverty, compared to 16.4% in Kentucky [30]. Approximately 57% of the county’s population lives in a low-population-density area [31]. Prior to and after the Covid-19 pandemic, unemployment in the county was higher (~5%) compared to Kentucky rates (~4%) [32]. Only 16.6% of adults have a bachelor’s degree or higher, compared to 27.8% statewide [30]. The health of Laurel County residents is also impacted by chronic health conditions, with 38.6% having obesity [33] and 10.4% reporting diabetes [34]. In 2021, food insecurity among the community was estimated as 14.8%, which was higher than Kentucky (12.9%) [35]. Further exacerbating food insecurity, the number of grocery stores in the county decreased by 35.3% (from 17 to 11 stores) in recent years [30].

Laurel County residents identified nutrition and obesity as top concerns, with the Laurel County Health in Motion Coalition identifying obesity as the #2 health priority behind substance use [36]. The Coalition identified multiple assets upon which healthy eating strategies might be implemented or replicated, including a farmers’ market and Extension programs. Notably, the top action steps recommended by the Coalition to reduce obesity were as follows: (1) encourage and provide social support interventions; (2) provide educational programs and presentations to school-age population on healthy nutrition; and (3) increase participation in programs providing healthy foods [36]. The community’s priority for improving food security and nutritional health outcomes provided a foundation for partnership between the community and university.

## 2. Methods

### 2.1. Building a Community–Academic Partnership

Collectively, the university research team had more than 50 years of experience implementing and evaluating community-based interventions, including projects collaborating with local Extension offices in Appalachia. The interdisciplinary team included experts in anthropology, nutritional science, epidemiology, behavioral science, and statistics across three academic units at the university. To begin building a partnership with the local community, the research team engaged with the Laurel County Extension office during the grant writing process. In addition, the research team gathered preliminary data from the community through a series of focus groups (*n* = 17 adult participants) (unpublished). Community members prioritized (1) more sources for buying safe, affordable, fresh local foods; (2) better skills for selecting and preparing food; (3) more healthy food options at restaurants and community events; and (4) more market opportunities for farmers and sustainability of family farms. Currently available assets included programming around healthy eating, but not all participants were aware of available programming. Participants indicated the most desirable intervention ideas included enhancing the availability of nutritious foods, improving education about the procurement and preparation of nutritious foods, enhancing the local farmers’ market, and increasing nutritious foods at food pantries.

In consideration of community feedback and input from the Extension office regarding community priorities, assets, and initial intervention ideas and costs, the research team outlined a community-engaged approach named Laurel HARVEST: Helping Appalachia Restore a Vibrant food Environment for Self Sufficiency Together. After the grant was awarded, the research team and the local Family and Consumer Sciences agent worked to form a CAB, convening individuals from diverse sectors of the community who had an interest in food security, agriculture, nutrition, and/or community engagement. The Institutional Review Board at the University of Kentucky approved procedures for this research project.

Eligibility criteria for the CAB included (1) residing in Laurel County for at least one year and no plans to move for the next three years; (2) over 18 years of age; (3) the ability to read, speak, and understand English; (4) willingness to share knowledge and opinions openly and honestly; and (5) willingness to meet at most two times per month until June 2023. Rationale for eligibility criteria included (1) identifying community members who have awareness of community resources and issues; (2) adults, as this was the target population for our study; (3) facilitating communication across members of the CAB and research team; (4) identifying people who will feel comfortable sharing concerns and ideas for the community; and (5) availability to regularly participate and provide feedback during the early phases of intervention selection.

Initial invitations for individuals to serve on the CAB were offered to representatives from the Extension Council (volunteers who support county Extension staff in identifying needs, setting goals, and delivering and monitoring programs that impact needs), 4-H (a youth development organization within Extension), the health department, nutrition education programs for low-income households (i.e., Supplemental Nutrition Assistance Program-Education and Expanded Food and Nutrition Education Program), food pantries, and the food bank. The Family and Consumer Sciences agent facilitated introductions between the project manager and local community members. Initial meetings included a project overview and goals, upcoming or current community health work, ideas and opportunities to collaborate within the county, and suggestions for other groups or people to invite to participate in the CAB. Meetings ended with an invitation to provide input on the direction of the project by participating in the CAB. After meetings with these community members, a snowball approach was used to identify more individuals who might be interested in participating in the CAB. Some community members took the initiative to share, in their own words, what they learned about the project with other community members and invite them to serve on the CAB, while others shared ideas of people for the project manager to contact.

Once a date was selected for an initial, informational meeting, letters, emails, and postcards were sent to formally invite people to attend. There was an open invitation to serve on the CAB for the duration of the four-year grant. When new groups or people showed interest, had resources to share, or were involved with interventions, they were invited to become part of the CAB and attend meetings. Records were kept regarding attendance at meetings.

### 2.2. CAB Operations

A project manager served as liaison between the research team and CAB members for day-to-day operations. The project manager worked with the Family and Consumer Sciences agent to organize quarterly meetings. Meetings were held either in person at the Extension office or via Zoom, whichever was most convenient for the CAB based on day of the week, time of day, and seasonal weather. Invitations and reminders were sent to all CAB members. Priority was placed on holding the first few meetings in person so that CAB members and the research team could build relationships over a shared meal [37].

The project manager prepared an agenda and took notes during each meeting. The meeting facilitator role was fulfilled by academic partners, either the project manager or one of the principal investigators. Meeting structure typically included introductions to accommodate new people and those who infrequently attended, a review of old business (e.g., project goals, updates for ongoing interventions), the presentation of new information, discussion and feedback, and the identification of action items before the next meeting. Meeting notes provided documentation of discussions, decisions, and action items.

### 2.3. Phases of CAB Work

#### 2.3.1. Community Assessment

The initial charge of the CAB was to identify available resources and barriers for accessing and eating nutritious foods for low-income households with children. Strategies included pulling secondary data from local, state, and national datasets, reviewing related scientific literature, and reviewing results of focus groups conducted with community members as part of the grant writing process [38]. CAB members were encouraged to share anecdotal and local data sources.

#### 2.3.2. Identification and Selection of Interventions

After the community assessment, the CAB prioritized the populations, barriers, and resources they wanted to focus on. The framework for increasing equity impact in obesity guided identification and selection of possible interventions to make nutritious options more available, reduce barriers to healthy eating, improve resources to support healthy eating, and/or enhance community capacity [39]. Additionally, there was an emphasis on planning interventions that involved both individual and policy, systems, or environmental approaches that could sustainably address top concerns [40]. The selection of interventions for the community required an iterative process that involved the CAB setting priorities, interim work by the academic partners to identify possible evidence-based solutions, reviewing options with the CAB, soliciting feedback and decisions from the CAB.

### 2.4. CAB Engagement

The CAB was charged with sharing their expertise by providing input on community priorities and the design, selection, and adaptation of interventions to promote access to nutritious food that are responsive to the community’s unique needs. The CAB also supported the selection of evaluation approaches, interpretation of findings, and dissemination of interventions and findings throughout the community. To engage the CAB between meetings, the project manager shared notes after meetings, provided updates, and asked for timely feedback as needed. Community members were compensated USD 15 via direct deposit for attendance at in-person and virtual meetings to offset time and/or travel.

To assess the experience of community members working with academic partners on this research project, community members were invited to anonymously complete a survey in December 2022, approximately three months after forming the CAB, and again in August 2024, two years after initiating the CAB. The decision to complete the first assessment three months after the CAB formed was based upon the desire for CAB members to have several meetings to reference when answering survey questions about interactions with each other and the research team, rather than a single interaction. The 23-item questionnaire included the brief version of the Research Engagement Survey Tool [41] and questions from the communication category of the Wilder Collaboration Factors Inventory [42]. The Research Engagement Survey Tool has two scales, one for quantity and one for quality of actions of partners leading the research. Each item utilizes a five-point scale, with 1 representing either never or poor and 5 representing always or excellent. Higher scores indicate higher engagement. Data were collected and managed using REDCap electronic data capture tools hosted at the University of Kentucky [43,44]. Additionally, a listening session was conducted with the CAB in August 2024, in which CAB members were invited to share qualitative feedback about their experience on the board, including what worked well, perceptions of factors that would make the project feel successful, concerns about the project thus far, and impact beyond this project. Informed consent was obtained from all participants for each assessment. The survey and listening session responses served as a formative process evaluation to guide the academic partners’ communication and collaboration efforts [45,46].

## 3. Results

### 3.1. CAB Composition

The project manager organized four meetings with local leaders to introduce the project and brainstorm a list of community members to invite participation on the CAB. To start, 27 invitations were extended for participation in the CAB. During the project, another four invitations were extended. The first CAB meeting was held in August 2022. In the first two years of the project, 17 people from Laurel County served on the CAB. Additionally, 11 members of the research team from the university attended meetings with the CAB. Community members represented Extension, farming, the food bank, food pantries, the school resource center, the health department, faith communities, and community resources for older adults. Of the 13 CAB members who completed the first engagement survey, members predominantly identified as female (77%, *n* = 10) and reported a mean age of 60 years (range 42–70 years).

### 3.2. CAB Operations

In the first two years of the project, the CAB convened 11 times. The frequency of meetings was slightly greater than originally planned due to challenges with identifying feasible policy, systems, or environmental approaches to improve access to nutritious foods. Most meetings (*n* = 8) were held in person at the Extension office. When meetings were held on Zoom (*n* = 3), there was a hybrid opportunity for people to join at the Extension office. During the first year of the project, in-person meetings lasted approximately 90 min. Over time, in-person and hybrid meetings required 60 min or less to work through the meeting agenda.

### 3.3. Phases of CAB Work

#### 3.3.1. Community Assessment: Historical Context and Community Priorities Related to Access to and Consumption of Nutritious Foods

The research team provided an overview of demographic, health, food security, and agriculture facts about Laurel County. The research team also reviewed results from focus groups conducted in the community as part of the grant writing process [38]. Focus group responses highlighted gains and losses of community resources during the pandemic, expansion and utilization of online food ordering, and diverse experiences regarding food access and preparation during the Covid-19 pandemic. The health department shared results from their 2022 Community Health Assessment that indicated that chronic diseases and access to more healthy choices were the top two concerns of the community. Additionally, approximately 25% of those who completed the health assessment survey indicated in the last three months they sometimes or often worried about food, reflecting food security concerns in the community [47]. Conversation during early CAB meetings shed light on historical and current efforts related to food gleaning and recovery efforts, fruit and vegetable voucher programs at the farmers’ market, and perceived barriers to healthy eating such as lack of awareness of available resources, cost, and lack of skills and time to plan and prepare meals. Collective input from the CAB generated a picture of a community invested in improving food access, food security, and nutrition-related outcomes. Early in the project, the CAB prioritized identifying interventions for working families, relatives raising children, and lower income households.

#### 3.3.2. Iterative Process to Identify and Adapt Multi-Level Community Strategies

**Figure 1:**
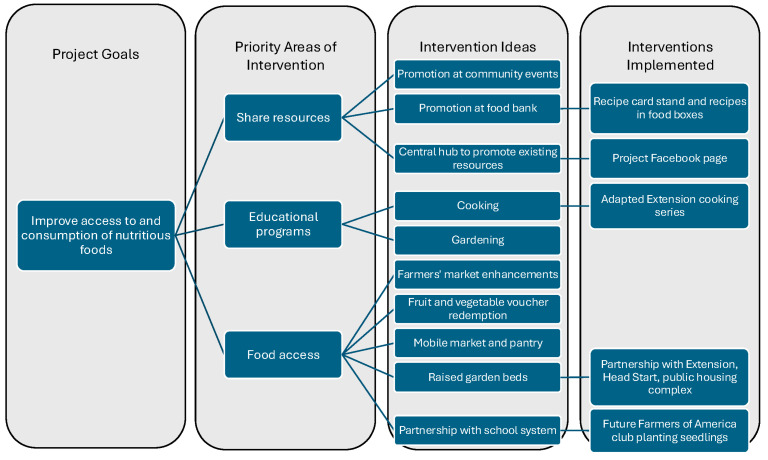
Overview of iterative process to identify, explore, and select multi-level community strategies to improve access to and consumption of nutritious foods.

Based on CAB priorities and discussions, some ideas were able to come to fruition while others were explored and unable to gain momentum due to challenges with feasibility, organizational capacity, and/or interest. Figure 1 provides an overview of the iterative intervention selection process.

#### 3.3.3. Family-Focused Educational Programs to Improve Skills and Resources for Cooking Nutritious Meals

A cooking class intervention was considered a quick win to improve consumption of nutritious foods, as the Extension office already offered a variety of cooking classes. Cook Together Eat Together, a “cooking social” approach for children and adults (i.e., families) to learn about planning nutritious meals and practicing cooking skills, is an evidence-based program currently offered throughout Kentucky Cooperative Extension [48]. To better meet the needs of families in this rural community, the program was adapted from eight in-person lessons to six lessons in which half were conducted in-person and half were conducted over Zoom. When conducted via Zoom, ingredient kits were provided so that families could make items at home. For each session, the CAB brainstormed and facilitated marketing of the cooking series to priority groups (working families, relatives raising children, and low-income households). Program evaluation surveys, options for participant focus group discussion, and check-ins with Extension agents after each session ensured the program continued to meet the community’s needs. Two years into the project, seven sessions of Cook Together Eat Together have been offered.

#### 3.3.4. Policy, Systems, and Environment Change to Improve Sharing of Resources

Other quick wins included sharing recipe cards from Extension at the food bank and creating a Facebook page. Recipe cards for distribution were selected based upon available produce and viewed as a way to promote consumption of foods people may be less familiar with. Facebook is an important resource in Laurel County. The Laurel HARVEST Facebook page served as an accessible hub to cross-promote existing resources and contribute to social norms in the community that support access to and/or consumption of nutritious foods.

#### 3.3.5. Policy, Systems, and Environment Change to Improve Food Access

Identifying one or more feasible policy, systems, or environmental approaches to increase access to nutritious foods took longer than anticipated (see Section 3.3.6 below), but a year and a half into the project, a connection was made between the research team and Head Start, a federally funded early childhood education program for low-income families, that led to collaboration with the Extension Master Gardener program to revive three raised garden beds and provide education at one Head Start Prep Academy site. Brainstorming sessions with the CAB led to the identification of three additional childcare centers and a public housing complex at which residents wanted to resurrect their community garden. The research team worked with each childcare center to set up and plant a garden table. The research team and Master Gardeners cleaned up and planted warm season plants (tomatoes, cucumbers, peppers) and cool season plants (cabbage, kale, cauliflower) in 9 raised garden beds at the 144-unit public housing complex. 

#### 3.3.6. Desired Policy, Systems, and Environment Change to Improve Food Access Not Feasible for Implementation

Early in the project, CAB members expressed an interest in leveraging the farmers’ market as an opportunity to increase access to nutritious foods. Barriers to consider for farmers’ market enhancements included transportation to the market, market hours, a decreasing number of local producers, cost at the market, and stigma associated with seeking and using food assistance program vouchers at the market. CAB ideas to address these barriers included a mobile market, expanded market hours, an additional market day, and increasing produce voucher and double dollar acceptance and redemption among older adults, participants in the Women, Infants, Children program, and/or families receiving Supplemental Nutrition Assistance Program benefits. Ultimately, potential solutions were viewed as unrealistic due to grant expense limitations (mobile market), concerns about decreased morning attendance needed to sustain the market (expanded market days and hours), challenges to find vendors to commit (expanded market days and hours and acceptance of vouchers), missed timelines (produce voucher programs), and resistance to systems change (accepting produce vouchers).

### 3.4. CAB Engagement

The first meeting included 15 community members and 3 academic partners. Over time, participation in the CAB fluctuated, and meeting attendance decreased. Meeting attendance by community members ranged 3–16 (median 8), and meeting attendance by academic partners ranged 2–6 (median 4).

#### 3.4.1. CAB Survey Responses

CAB members who completed the survey perceived high engagement between community and academic partners at the beginning of and two years into the project (Table 1). CAB members indicated high frequency and quality actions of research partners to build trust, feel individual work is valued, feel that opinions matter, build on strengths and resources in the community, and demonstrate mutual respect. While the scores of the quality of actions of research partners slightly decreased between time points, scores remained high.

Twelve CAB members completed the section of the survey about communication efforts at the beginning of the project, and seven completed it two years into the project. Mean responses for each of the five questions, at both time points, were 4.0 or higher, out of 5, indicating communication within the CAB was a strength. Overall, survey responses indicated no obvious concerns from CAB members about interactions with academic partners.

#### 3.4.2. CAB Listening Session Responses

Conversations during the listening session reinforced satisfaction with communication and identified the strengths of working as a community–academic partnership and the benefits of being part of the CAB. CAB members felt there was a good dynamic in the group, meaning members enjoy one another’s company, and that creates a meeting environment that feels more like a conversation in which people want to participate. A real strength was the bidirectional opportunity to create and explore intervention ideas. Ideas generated at meetings gave academic partners and individual members ideas they may not have thought of otherwise. For example, after learning about food recovery programs, one individual instituted a gleaning program with a local grocery store to obtain vegetables for older adults. Additionally, the grant support provided resources that allowed local organizations to extend their work and do things they would not otherwise be able to achieve.

The CAB members identified three outcomes that would make the project successful: (1) creating partnerships; (2) improving and expanding reach of resources; and (3) sustaining raised garden beds to bear produce over time. The CAB members liked the flexibility of the group, the responsiveness of the academic partners to the community’s ideas, and the willingness to adapt ideas to accommodate input. Additionally, CAB members said participation in the project thus far has provided useful networking opportunities across the CAB membership and broader community, which allowed for increased knowledge about and sharing of resources. They recognized the hard work everyone put into the project and valued the opportunity to learn about and share community resources—“If we can’t do it, we find someone who can”, one CAB member remarked. The candid conversation facilitated ongoing brainstorming and conversation about working with new groups, prioritization for interventions and next steps to achieve the project goals. Ideas included advertising project successes through local and social media, aligning the project with large community events, and working with high school and home school students interested in horticulture, agriculture, and/or youth development.

## 4. Discussion

Addressing obesity prevention and food insecurity in rural communities remains a challenge [49,50], particularly considering high food prices [51], and community–academic partnerships provide a key approach for identifying, adapting and evaluating multi-level strategies [51]. Understanding factors that nurture or hinder such partnerships is important for assuring the success of such strategies [52], and our evaluation of the process for Laurel HARVEST provides insights into key elements that foster advancing such partnerships.

Bringing the “right” group of people together can facilitate a community–academic partnership [52]. Within the initial process of identifying individuals to serve on the CAB, Cooperative Extension was a vital informant for recognizing members and organizations within the community that are involved in the space of healthy living and food security [22]. Each member of the CAB contributed unique interests and talents to the group as well as insights across the community’s food system. Though we began with a group of 17 individuals from Laurel County, attendance and participation remained at approximately 8 individuals at each meeting. It is not uncommon for CAB members to move on from active status within the group or have fluctuating attendance throughout research projects, depending on their personal and professional obligations [53,54]. We offer the following speculations for the change in participation. The composition of CABs, by nature, is fluid. As the focus and needs of the project evolved, so did the contributors. Each CAB member participated in additional organizations throughout the community and may have felt that this project added more to their plate or that the ask outweighed the benefit. The timing of meetings, initially agreed upon, may have become less convenient. Regardless of attendance, we continued to share invitations to and summaries of meetings, leaving open the opportunity to rejoin at their convenience. In the future, we will explore the impact of sending more personalized invitations to previous attendees. Among those who agreed to be on the board, we noticed strong buy-in from those who had an active, or long-standing role in non-profit organizations or the community. Notably, of the community members who attended more frequently, several had served the community for years in organizations or activity areas such as local food pantries, churches, or recognized the value of Extension services to provide education to their community. Likewise, of those who attended more frequently, we saw cross-relationships of organizational efforts outside of the CAB membership. Some members were able to make new inter-group connections from their time with the CAB and foster new activities throughout the community, potentially suggesting that the quality of relationships, trust, and community impact among CAB members is more important than the number of CAB members [19,52].

The process of identifying, evaluating, and selecting interventions, as outlined for the Laurel HARVEST project, underscores the critical iterative nature of CAB engagement. Throughout the project, meetings with CAB members were efficient and fruitful in terms of idea generation for programs or initiatives to achieve the Laurel HARVEST goals. Bringing together residents that possess deep understanding of community history, values, norms, and assets with investigators allowed for thoughtful discussions about which interventions would be acceptable to the priority populations. While investigators were pleased to implement some interventions as suggested by the CAB, other interventions required adaptations, and some interventions were not feasible due to cost, time, or other barriers. It was important for us, as researchers, to foster idea generation and allow space for the CAB to continue to have big goals even though all may not be feasible. Although some ideas were unable to be brought to completion due to various factors, partners practiced diligence in exploring each option, which likely contributed to the building and maintaining of relationships, and explaining why certain interventions (e.g., farmers; markets enhancements) were not feasible helped to build trust between CAB members and researchers [55]. Additionally, discussion of barriers for interventions with the CAB fostered shared understanding and a bidirectional co-learning process [56]. In some instances, when an intervention seemed an unlikely option, our discussions revealed potential modifications to an intervention that would uphold the rigor of the intervention while creating a better fit for the community (e.g., garden tables instead of raised beds at child care settings). These experiences of due diligence and transparent communication may translate to others attempting to implement interventions in resource-constrained settings. Other tactics that have been found to foster successful community–academic partnerships to address health inequities include engaging key stakeholders in the planning phases of the research [57] and attention to group dynamics [58], both of which were integral to Laurel HARVEST.

Based on survey feedback, CAB members reported small reductions in the quality of the relationship between themselves and university partners. Overall, there was a smaller number of participants in the second iteration of the survey and within the “quality of action” portion of the survey. Likewise, due to the anonymous nature of the survey, CAB members completing the survey at each time point may not have been the same. The first CAB survey was conducted in December of 2022, which was within the first few months of the project, while the second follow-up survey was approximately 2 years later. Due to the initial survey being collected earlier in the community–academic partnership, the second iteration may have offered a more realistic insight into perceived quality of the bi-directional relationship following more time together and communications. Another consideration for lower perceived quality of action may be due to CAB members feeling as if the amount of progress made did not meet their expectations after approximately two years of work. Though not explicitly mentioned by the CAB, which may be due to the nature of the Likert-style questions being asked, it may be recommended to additionally ask CAB members to complete anonymous, open-ended questions to allow for more investigation into their rankings of quality of action by the research team.

This description of Laurel HARVEST offers an in-depth example of the process of building a community–academic partnership with a rural community to identify multi-level interventions to improve food security and nutritional health outcomes for low-income populations. The ongoing assessment of engagement and function of the CAB through a combination of quantitative, anonymous surveys and open-ended conversations with CAB members provided critical information for guiding future interactions with the CAB. However, our findings are subject to several limitations, including responses that were self-reported among a small group of CAB participants, representing a purposive sample. Examples of bias that may have resulted from this method of data collection include social-desirability bias and response-shift bias, both of which may have decreased or increased over the course of the study. The use of validated surveys and the inclusion of listening sessions as a secondary source of data provided some rigor against this. Future strategies to mitigate or assess the impact of bias could involve third-party observation during CAB meetings and survey input from academic/research team members. Anonymous surveys prevented matching responses to CAB members at the two time points, which precludes us from the use of inferential statistics to determine any statistically significant changes over time. Additionally, the use of snowball sampling to purposively recruit CAB members may have unintentionally excluded less connected or underrepresented community members who would have offered valuable contributions. Lastly, these results have limited generalizability beyond the context of Laurel County. Findings may be most applicable to other rural, Appalachian communities that have similar sociodemographic profiles and relatively robust interest in, and existing resources for, supporting access to and consumption of nutritious foods. However, our methods and results may be helpful to others seeking to build community–academic partnerships in rural settings.

## 5. Conclusions

CABs provide an opportunity to build meaningful, productive relationships between rural communities and academia that allow representatives from the community to guide the development and implementation of interventions that leverage a community’s resources to address its priority needs. Full exploration of potential solutions suggested by community members may build trust between community and academic partners and can provide a strategic selection process for multi-level, evidence-based interventions deemed feasible for addressing complex issues such as food insecurity and nutritional health outcomes.

## Figures and Tables

**Table 1:** Community CAB members’ perceived quantity and quality of engagement with academic research partners [39].

	Quantity of Action of Academic Partners Leading the Research (1 = Never; 5 = Always)		Quality of Action of Academic Partners Leading the Research (1 = Poor; 5 = Excellent)	
	December 2022 (*n* = 13)	August 2024 (*n* = 8)	December 2022 (*n* = 10)	August 2024 (*n* = 4)
Focus is on problems important to community.	4.8	4.8	5.0	4.8
All partners assist in establishing roles and related responsibilities.	4.8	4.8	4.6	4.5
Community-engaged activities continue until the goals are achieved.	4.7	4.8	4.8	4.3
Partnership adds value to work of all partners.	4.8	5.0	4.8	4.5
Team builds on strengths and resources within the community.	4.8	4.8	4.7	4.5
All partners’ ideas are treated with openness and respect.	4.8	4.9	4.9	4.7
All partners agree on timeline for making shared decisions.	4.8	4.5	4.8	4.0
Partnership processes support trust among all partners.	4.8	4.8	4.8	4.3
Mutual respect exists among all partners.	4.9	5.0	4.9	5.0

## Data Availability

The raw data supporting the conclusions of this article will be made available by the authors on request.
